# Education of pharmacists in Ghana: evolving curriculum, context and practice in the journey from dispensing certificate to doctor of pharmacy certificate

**DOI:** 10.1186/s12909-020-02393-x

**Published:** 2020-11-26

**Authors:** Augustina Koduah, Irene Kretchy, Reginald Sekyi-Brown, Michelle Asiedu-Danso, Thelma Ohene-Agyei, Mahama Duwiejua

**Affiliations:** 1 Department of Pharmacy Practice and Clinical Pharmacy, School of Pharmacy University of Ghana, P. O. Box LG43, Legon, Ghana; 2 Department of Pharmacology and Toxicology, School of Pharmacy University of Ghana, P. O. Box LG43, Legon, Ghana

**Keywords:** Dispensers, Educational reforms, Ghana, Nurse-dispenser, Pharmacist, Pharmacy education

## Abstract

**Background:**

Understanding the origin and evolution of education of pharmacists is important for practice and health system reforms. In Ghana, education of pharmacists started in the 1880s with the training of dispensers in a government hospital. Over the years, the curriculum and institutional arrangements changed and currently pharmacists are trained in universities. In this study we explored how and why education of pharmacists evolved in Ghana.

**Methods:**

We used a case study design to systematically describe education of pharmacists reforms. Data was collected from October 2018 and December 2019 through document review and in-depth interviews. The data was analysed based on institutional arrangements and contextual factors influencing reforms from the 1880s through 2012, when the Doctor of pharmacy programme was initiated in Ghana.

**Results:**

Reforms occurred around four main periods when institutional arrangements including the certificate awarded and expected roles were modified by educators and government. These are: (1) the Certificate of dispensing with dispenser-in-training and nurse-dispenser schemes (1880s to 1942), when dispensers were trained to assist doctors in dispensing or directly diagnosing and treating specific disease conditions. (2) the Diploma and Certificate of competency with the dispenser-in-training and pupil pharmacist schemes (1943 to 1960), where in addition to existing roles, pharmacists operated village dispensers. (3) the Bachelor of pharmacy degree (1961 to 2017), when pharmacists were trained mainly as medicines experts with a strong science base on all aspects of medicines from production, distribution and use; and over time with a gradual move to patient-oriented practice. (4) the Doctor of pharmacy degree (2012 to date), where in addition to existing roles, trainees are exposed to advance professional practice experiences. Important factors influencing the reforms included, health systems demands for village dispensaries and clinically oriented pharmacists, and harmonization with regional and international training and practice.

**Conclusion:**

Reasons influencing education of pharmacists reforms are context specific and are driven by historical experiences, national and international expectations as well as educators and regulators abilities to influence change. These reforms call for direct corresponding change in professional practice laws and regulation to enable pharmacists contribute fully to health care delivery in Ghana.

## Background

‘*A pharmacist is a scientifically-trained graduate healthcare professional who is an expert in all aspects of the supply and use of medicines. Pharmacists assure access to safe, cost-effective and quality medicines and their responsible use by individual patients and healthcare systems’* [1]. Pharmacy practice is dynamic and the context in which this definition is interpreted and applied is determined by national priorities, education and health systems.

In Ghana, the education of pharmacists has evolved over a century beginning with the training of dispensers in government hospitals in the 1880s (then the Gold Coast) through apprenticeship [2] to present, where training institutions are university-based with experiential experiences offered in mainly the hospitals, pharmaceutical industries, pharmacy and medicines regulatory institutions and community pharmacies. Certificates awarded have correspondingly transitioned from Certificate of dispensing, Diploma and Certificate of competency, Bachelor of pharmacy degree (BPharm) to Doctor of pharmacy degree (PharmD). Each level of certification had responsibilities commensurate with health system requirements, however, regulatory laws for professional practice has not always been in synchrony with the competence associated with the training.

Globally, pharmacy education has transformed from the traditional drug-focused training to a more patient-focused education, with the goal of optimizing patients’ medication-related problems to achieve definite outcomes. Requirement to practice as a pharmacist has accordingly transformed with most countries defining entry-level qualifications into the profession as either Master of pharmacy degree (MPharm) as in most European countries or Doctor of pharmacy (PharmD) as in USA and many other countries [3].

The drivers of change for education of pharmacists are varied and context specific. These may include changing societal needs and expectations, regulatory standards for education, and national, regional and international policies [4, 5]. The deliberate efforts and decisions taken by educators, regulators and government to constantly revise training curricula and institutional arrangements to reflect governing standards for training and practices, health systems needs and expectations as well as national and international policies is therefore critical for learning and better understanding of the drivers of these reforms.

There is limited research on how education of pharmacists in the Ghanaian context started and evolved and the reasons for such change. Related documentation in the literature have focused on history of medical services in Ghana [2], practice of pharmacy in Ghana [6], pharmacy professionalism in Ghana [7] and clinical pharmacy training in Ghana [8]. In this paper, we systematically describe how and why education of pharmacists started and evolved in Ghana. We argue that contextual factors and policy actors such as educators and regulators greatly influenced these reforms and the practice.

## Methods

### Study design

A longitudinal case studies of reforms for education of pharmacists was conducted for the period 1880s to 2012. The case study approach was appropriate since it allowed the use of multiple sources of evidence and triangulation for in-depth investigation within a real-life context to trace change and provide explanation of the phenomenon [9]. We attempted to systematically reconstruct these historical and contemporary reforms focusing on when, where and why education of dispensers (as they were known in the 1880s) started and how entry requirements, curricula, expected roles and certificates awarded, had evolved over time. Data was collected between October 2018 and December 2019 using extensive desk review and analysis and in-depth interviews.

### Data sources and collection

The study relied greatly on reviews and analysis of documents and archival materials including colonial correspondence and reports (Table 1) to map historical and current happenings. The documents and archival materials were assessed to ensure that the evidence is original, free from errors, typical, clear and comprehensible [10]. The colonial correspondence, medical department reports, Ordinances and Acts on pharmacy practices, reports on medical education, pharmacy curricula and experiential learning manuals were retrieved from Ghana’s Supreme Court, Ghana Publishing Corporation and University of Ghana Balme Library. Published literature related to education of pharmacists and pharmacy practice were obtained using a web-based search engine google scholar.

**Table 1:** List of documents and archival materials

Documents and archival materials	Year
**Colonial letters and correspondents**	
Correspondence relating to a scheme for extending the system of village dispensaries and improving the training of the African nursing and dispensing staff (No. XV of 1930–31) Gold Coast. Despatch from the Governor to the Secretary of State (N. 389)	2 June 1930
Letter from Medical Department to the Colonial Secretary. Subject: Respecting appointment of medical tutor in connection with extension of village dispensaries and the improvement of the training of the native nursing and dispensing staff.	2 September 1930
Letter from Medical Department to the Colonial Secretary. Victoriaborg, Accra No. 150/33/14 (Ref, 348/30/S.F.1). Subject: Nurse-Dispenser Scheme	27 March 1934
Letter from Colonial Secretary to the Director of Medical Services (Ref. 348/30/S.1/169)	19 September, 1939
Letter from Medical Department to the Colonial Secretary (No.150/33/147)	13 April 1939
**Reports**	
The training of nurses and dispensers for employment in government services- revised scheme, including a revised scheme for village dispensaries. A report from Medical Department to the Colonial secretary	10 April 1930
Response to report of the committee appointed by Secretary of State in 1927, which dealt with scheme for the creation and training of an auxiliary services of medical assistants. By Act Governor -SGD. G.C. Du Boulay	2 June 1930
Sessional Paper XV of 1930–31 (File No. 150/33)	1930
Extract from Report of Select Committee on Estimates, 1934–35	21 March 1934
Extracts from Report of Select Committee on Estimates, 1937–38 (File No. 192/37)	1937
Extract from the report of select committee on the estimates, 1939–40 (File No.348/30)	1939
Nurse-Dispenser Scheme (No.150/33/147). From Medical Department to the Colonial Secretary Office:	13th April 1939
Requirements in Grade of 2nd Division Nurses and Nurses-in-Training (File No. 150/33) (Notes brought by Acting Director Medical Sanitary Service for discussion on 18 May 1934)	1934
Notes of discussion with Acting Director Medical Sanitary Service	18 May 1934
**Ordinances and Acts**	
Drugs and Poisons Ordinance No. 14 of 1892 (Druggist Ordinance)	1892
Pharmacy and Poisons Ordinance CAP.70 of 1946	1946
Pharmacy and Drugs Act 64 of 1961	1961
Pharmacy Act 489 of 1994	1994
Health Professions Regulatory Bodies Act 857 of 2013	2013

Data from the documents and archival materials were mapped based on these guiding questions: When did education of pharmacists start in Ghana and how did it evolve and why? What entry criteria were required and what courses were taught? What certificates were awarded, how and why did it change over time? What contextual factors were important in promoting the decision-making process of change and why? Which policy actors were involved in the decision-making processes and what were their roles in the process?

Key informant interviews were based on same questions. Five key informants with historical account and experiences of decisions relating to education of pharmacists were purposively selected based on availability and interviewed to better understand the nuances from the extensive documents review and analysis. Our key informants included a former educator (founding dean of the school of pharmacy, University of Ghana); two former Pharmacy Council Board Chairpersons (1994–2009, 2009–2015), a past president of the Pharmaceutical Society of Ghana (2011–2015) and a past registrar of the Pharmacy Council (2005–2017). These key informant interviews allowed sharing of experiences of direct participation and observation of the issue under study [11]. Interviews lasted 45 min on average. In total six interviews were conducted between 28 November 2018 and 3 May 2019. Interviews were tape recorded and later transcribed verbatim. Where permission was not granted to record, notes were taken and verified later with the respondents. Respondents were informed of the study purpose and verbal consent sought. Interviews stopped when respondents provided no new information. The transcriptions were analysed and organised into retrievable sections based on the research questions.

### Data analysis

Entry requirements, curricula, and certificate awarded were traced and mapped from when they were first documented in the 1880s to 2012 when the PharmD programme was initiated. First, the entry criteria and training institutions were noted. Specific courses taught and expected roles of pharmacists in the healthcare system and how these differ over time were documented. For each period, certificates awarded, specific courses, the titles assigned to pharmacist trainees and the reasons for change were mapped, categorized and compared.

Further analysis involved mapping the contextual factors and policy actors relevant in promoting changes in decisions relating to the title assigned to pharmacist trainees, nature of certificates awarded, and their expected roles in the Ghanaian healthcare system. We drew on Grindle and Thomas’ conceptualization of policy actors and their context [12] to map and categorize contextual factors influencing policies relating to education of pharmacists in Ghana. They propose that within decision making situations to change existing policies and practices, policy actors operate within several interlocking contexts that confront them with problems they need to address and set solutions that are workable not only administratively but also politically and economically. Policy actors therefore do not work in vacuum and are never fully autonomous. They defined context to include historical experiences and conditions, domestic economic conditions, international and political relationships, administrative capacity of the state, and the impact of prior or conterminously pursued policies [12]. We therefore mapped policy actors and their role as well as contextual factors that influenced decisions relating to education of pharmacists in Ghana. We acknowledge the challenges in chronologically mapping contextual factors influencing decisions dating back to the 1880s. We used varied data sources and triangulation as ways to deal with the challenges.

### Ethical consideration

Ethical approval to conduct this study was secured from the University of Ghana College of Health Sciences Ethical and Protocol Review Committee with protocol identification number: CHS-Et/M.10-PI.1/2017–2018 and approved on August 3, 2018. As per the ethics, verbal and written consent were attained from respondents before commencement of interviews.

## Results

Education of pharmacists in Ghana started in the 1880s (then the Gold Coast) and evolved along four main periods when the trainee titles, schemes and certification changed. The periods were (1)1880s to 1942 - Certificate of dispensing with the dispenser-in-training and nurse-dispenser schemes; (2) 1943 to 1960 – Diploma/Certificate of competency with the dispenser-in-training and pupil pharmacist schemes; (3) 1961 to 2017 - Bachelor of pharmacy degree and (4) 2012 to date - Doctor of pharmacy degree. These periods occurred under varied contextual factors that influenced the reforms. During these periods, dispensing and pharmaceutical care evolved. Historically, care focused on dispensing prescription, compounding, mixing of drugs and poisons with later nursing and midwifery, drug oriented care and then patient oriented care.

### Certificate of dispensing (1880s-1942)

#### Trainee title: dispenser-in-training (1880s-1930)

In the colonial era, healthcare systems focused on health provisions for the Europeans who were in the Gold Coast at the time. The indigenous people begun to access public health facilities when the government built the first civil hospital in Accra (1878). As a result, by the early 1880s, individuals with minimum educational background of Standard VII school certificate [13] were trained by the government as dispensers to assist medical officers [7]. They were trained in the Accra Hospital for three years through practical training and apprenticeship under medical officers [2]. Courses taught were dispensing and basic nursing. After training, the students were awarded certificate of dispensing and employed by the government as dispensers.

To streamline the practice of persons retailing, dispensing or compounding drugs and poisons, the government enacted the Drugs and Poisons Ordinance No. 14 of 1892. The Ordinance set a precedent for all dispensers to be examined by a Board of Examiners (regulatory body) created under the Ordinance. Candidates for licensure examination were to provide one of the following: (1) ‘*a certificate of employment in compounding and dispensing of prescriptions in a colonial hospital in the Colony or any other of Her Majesty’s colonies or dependencies for three years’,* (2) ‘*a certificate of employment in the compounding and dispensing of prescription as an assistant to a duly qualified medical practitioner, apothecary, or chemist and druggist, for a period of five years’* and (3) ‘*satisfactory evidence that for a period of three years preceding the commencement of this Ordinance he has been engaged in the colony in the selling, compounding and dispensing of prescriptions in some house or shop kept by him for the purpose’*. Under the Ordinance, a register of all duly examined dispensers was kept and each dispenser issued a licence to practice as a druggist [14].

By 1907, there were 18 dispensers and 16 dispenser-in-training in country. Few individuals were trained as dispensers because fewer indigenous people went to school [2]. Additionally, the salary of the dispensers was minimal. In 1900, a senior dispenser was paid about £50 and a newly qualified medical officer paid about £400. This minute salary dissuaded indigenous people from taking up the training. By the early 1910s, some dispensers in government hospitals had left and opened their own drug stores. In 1912, the Medical Department improved conditions of services for dispensers and other supporting staff to attract individuals for training and for them to stay on the job after training [2].

Governor Clifford in the early 1910s with the support of the principal medical officer Dr. Rice, introduced a policy to expand medical service to indigenous people through a dispensary system. Within this context, the government promoted village dispensaries as a cost-effective way to expand healthcare to the indigenous people. Government initiated the process to train more dispensers to operate village dispensaries under the supervision of medical officers and by 1919, there were 23 dispensers in training [2, 7, 15].

To restructure and amplify the training of dispensers, the dispensing school established in the Accra Hospital was moved in 1925 to the Korle Bu Hospital. Teaching was done in the wards, out-patients clinics and in the dispensing department [2, 7, 16]. With the dispensing school in Korle Bu Hospital, suitable candidates with Standard VII certificate were recruited twice yearly at the end of January or July immediately after the Druggists’ examination and were on probation for three years. Dispenser-in-training were not paid for the first 18 months of their programme, but later put on pay roll upon recommendation by their instructors [17].

Under the restructured training for dispensers the curriculum was detailed and extensive. In the first year, there was three months of lectures, reading and progress examinations in anatomy, first aid and surgical nursing; and nine months of ward training. During the ward training, students attended lectures, undertook same duty activities and examinations as nursing students. However, would-be dispensers were trained in anaesthetics. In addition, the matron and resident medical officer sent regular reports on each dispenser-in-training student to the dispenser’s instructor. In the second year, the students were taught elementary chemistry and materia medica - the various drugs in the British Pharmacopoeia i.e. appearance, taste, odour, uses, doses, incompatibilities, mode of preparation, weights, measures, symbols, prescriptions etc. Theory-based courses were supplemented by practical demonstrations in the Pharmacy room, and a progress examination held every week. In the final year, students were taught therapeutics, anaesthetics, and poisons (characters, dangerous doses, symptoms, antidotes and remedial measures) [17]. The training equipped the dispensers with knowledge and skills to diagnose and treat specific and common disease conditions such as malaria, diarrhoea and yaws in addition to the roles of dispensing and compounding of drugs [18].

#### Trainee title: nurse-dispenser (1931–1939)

By the late 1920s, requests by chiefs (traditional leaders) for dispensaries in their villages had increased and the existing numbers of dispensers were inadequate to meet the demand. Nurses on the other hand, could not adequately manage the village dispensaries because they were untrained in dispensing [18]. Therefore, the Secretary of State in 1930 introduced a policy, the “nurse-dispenser” scheme to increase training of officers for the village dispensaries. The scheme had already been implemented in Sierra Leone, another British colony [15]. The scheme combined nursing and dispensing to produce individuals to meet the needs of the projected system of village dispensaries. The dispenser-in-training course was therefore, improved to include elementary training in midwifery and sanitation [17, 18]. The intent of the nurse-dispenser scheme was to train an officer who could not only run a village dispensary but also act as a dispenser in a general hospital as well as a nurse if need be [19].

With the nurse-dispenser scheme in place, dispenser-in-training trainee title was abolished, and all prospective dispensers were enlisted as nurse-in-training in the first instance. Standard VII education remained the entry requirement and all students joined as nurses-in-training and spent the whole of the first year in the wards. During the second year, they spent one hour per day in the dispensing school and the reminder of the time in the wards. In the third and fourth years, students alternated between the dispensing school and the wards. At the end of three years, students sat for examination in nursing and when they passed, they were classified as 2nd division nurses. Table 2 summarises the number of students in training at the dispensing school and expected year of qualification as a nurse-dispenser between 1934 and 1938 [18].

**Table 2:** Number of students and their year of completion under the Nurse-Dispenser Scheme

**Year**	1934	1935	1936	1937	1938
**No. of students qualifying**	6	11	7	9	17

As 2nd division nurses, they worked as before by alternating in the wards and in the dispensing school till they sat for the Druggist examination as required by the Druggists Ordinance [14, 19]. Students who passed the Druggist examinations were classified as nurses until a dispenser position was vacant in the government hospital or village dispensary [15]. To promote males as dispensers (druggists), the grades for chief nurse and 1st division nurse were reserved for females. All senior positions for males above the grade of 2nd division nurse were held by dispensers, but they functioned in the capacity as nurses or dispensers depending on the requirement of the service [15]. The distribution of males and females over the different functions were not stated in the documents reviewed. However, estimated requirement for 1934–1935 were gender specific. For example, 50 males and 19 females for the hospital and 20 males and 10 females as staff in training were estimated for the Colony [20].

*‘In the 1930s and 1940s, brilliant male nurses who were trained as dispensers worked in village dispensaries and some had their own drug stores’* (Former Pharmacy Council Board Chair:14/12/2018).

### Certificate of competency/diploma (1943–1960)

#### Trainee title: dispenser-in-training (1940–1945)

Under the nurse-dispenser scheme most trained individuals proceeded as druggists, creating a deficiency in the number of practicing nurses. The Medical Department therefore, in 1939 reverted to the old system of recruiting and training nursing and dispensing staff separately [19]. In the revised scheme, Cambridge School Certificate (with passes in biological sciences, physics and chemistry) was required for admission in addition to passing an entrance test [6]. Students were trained for three years and awarded a diploma.

The training was revised to include physiology, pharmaceutical technology, practical pharmaceutics and advanced dispensing (i.e. preparation of liniments, granulation and tablet making). However, an aspect of the nurse-dispenser scheme was maintained; qualified dispensers worked in the wards for 6 months as supernumeraries [6, 19]. A dispenser had to pass the licensure examination under the Druggist Ordinance to practice as a druggist [14, 19].

#### Trainee title: pupil pharmacist (1946–1960)

In 1946, the Drugs and Poisons Ordinance No.14 of 1892 was repealed and replaced with the Pharmacy and Poisons Ordinance No. 21 of 1946 [21]. The regulator - Pharmacy and Poisons Board prescribed courses of instructions for pupil pharmacists and issued certificate of competency to pupil pharmacists who passed licensure examination and had satisfactory evidence of good character [21]. The Pharmacy and Poisons Ordinance introduced the term ‘pharmacist’ and by 1947, all persons registered and licensed under the Druggist Ordinance of 1892 were also designated as pharmacists [6].

*‘The title pharmacist was already used in Britain and its introduction in the Gold Coast uplifted the pharmacy profession’* (Former Pharmacy Council Board Chair:14/12/2018).

In 1951, the dispensing school was relocated to Kumasi to be part of the Kumasi College of Science and Technology as a Pharmacy department [6, 7]. The Pharmacy department subsequently revised the existing pupil pharmacist curriculum. Students were no longer trained in nursing and working in wards for 6 months as supernumeraries was discontinued.

‘*When the dispensing school moved to Kumasi all forms of nursing training were stopped and the focus was to align to the training of pharmacists in other settings’* (Former Pharmacy Council Board Chair: 3/12/2018).

By the early 1950s government had to train male nurses or pharmacists as clinical superintendents to run the village dispensaries (now health centres) under the supervision of district medical officers to fill the gap that the cancellation of the nurse-dispenser scheme had created [2]. In 1950, only 22 out of over 100 village dispensaries were run by pharmacists. Over time, the role of pharmacists in clinical service delivery reduced and their role as a dispenser-nurse disappeared from the 1960 National Health Development Plan (Ghana gained independence in 1957) [2]. Additionally, the Medical and Dental Act, 1959 (No. 36) restricted the right to practice medicine or dentistry and to recover charges to only medical professionals [22]. Therefore, pharmacists were legally unable to operate the village dispensaries. However, pharmacists were allowed by the Pharmacy and Drugs Act, 1961 (64) ‘*to give medical or dental advise or aid by way of first aid in the case of accidents; or by way of first treatment in the case of simple ailments of common occurrence where it is not reasonably practicable for the patient to consult a medical practitioner or dentist, as the case may be’* [23].

### Bachelor of pharmacy degree (1961–2017)

In 1960, the Kumasi College of Science and Technology gained a university status as the University of Science and Technology and this gave way to a four-year degree course in pharmacy and the award of a Bachelor of pharmacy degree [6, 7]. A pass in Advance Level Certificate chemistry, physics, biology, mathematics and general paper was a requirement for entry. The existing diploma curriculum was revised, and the degree course aligned to what pertained to the training of pharmacists in the United Kingdom. The course included pharmaceutical sciences, pharmaceutics, microbiology, pharmaceutical chemistry, pharmacology and pharmacognosy. These courses were offered at different stages during the four-year programme and the students were trained to be experts and advisers on drugs [6]. Upon completion of the degree course, students undertook practical training (internship) as stipulated by the Pharmacy and Drugs Act, 1961 to qualify for a licensure examination [23].

*‘In 1963 the certificate of competency/diploma was cancelled in the University of Science and Technology’* (Former Pharmacy Council Board Chair: 3/12/2018).

Individuals with certificate of competency issued under the Pharmacy and Poisons Ordinance [21] or licensed and registered under the Druggists Ordinance [14] were allowed to practice as pharmacists under the Pharmacy and Drugs Act 64 [23]. In 1994 when the Pharmacy and Drugs Act 64 was repealed and the Pharmacy Act 489 enacted, a degree in pharmacy became the required qualification for licensure examination [24]. Overtime, the number of newly registered pharmacists increased as the number of students intake increased.

By the early 2000s, the courses were revised, and the curriculum included pharmaceutical chemistry, physiology, biochemistry, pharmaceutics, microbiology, chemical pathology, pharmacology, applied therapeutics and pharmacy practice to meet national and international expectations and demand. Though training of pharmacists as experts on drugs and less emphasis on clinical experiential learning still persisted, the Ghanaian healthcare system and the general public demanded more clinical expertise from pharmacists [8] thus demanding some elements of the 1930 nurse-dispenser scheme.

Additionally, there was a global drive to upgrade education of pharmacists and train patient-oriented pharmacists. A World Health Organization (WHO) meeting on revision of undergraduate pharmacy curricula held in Nyanga, Zimbabwe (18–20 April 1997) recommended focus on patient-oriented pharmacy practice by introducing courses such as hospital pharmacy, community pharmacy, clinical pharmacy and pharmaceutical care [25]. The Faculty of Pharmacy therefore in October 2001 established a department of Clinical and Social Pharmacy to introduce courses in clinical pharmacy, social pharmacy and public health patient-oriented practice. From June 2003 students in their third year started rotations at various health care facilities for experiential learning [8]. The duration of training remained four years with a strong drive towards the teaching of pharmacy practice.

The University of Science and Technology now Kwame Nkrumah University of Science and Technology (KNUST) was the only public university in Ghana awarding a degree in Pharmacy, until 2007 when the School of Pharmacy in University of Ghana (UG) was established as well as a private school of pharmacy (Central University). The number of registered pharmacists increased gradually over the years.

### Doctor of pharmacy (2012 to date)

*‘Pharmacy education had evolved globally with countries such as the USA, Algeria, Thailand and Nigeria training PharmD students and there was the need to upgrade’* (UG School of Pharmacy former Dean: 30/03/2019).

*‘Nationally, there were increased demands for patient-oriented pharmacists with skills for patient care needs’* (Pharmaceutical Society of Ghana past President: 18/04/2019).

With the early revisions in the BPharm curriculum by pharmacist educators towards clinical and pharmacy practice courses, the introduction of PharmD was a move towards addressing this need while expanding the role of the pharmacists in the Ghanaian healthcare system. An additional driver for the transition from BPharm to PharmD was the resolve of the Economic Community of West African States (ECOWAS) ministers of Health, through its agency the West Africa Health Organization (WAHO) to remove disparities in content and harmonise health training in the region to facilitate movement and sharing of health personnel across the region. This was seen as a solution to the brain drain and shortages in health workforce within the region. Having agreed that the four year Bachelor of Pharmacy degree could not adequately accommodate the new competencies required of pharmacists in the region. The deans of Pharmacy opted for the six years Doctor of Pharmacy Programme. This decision was also more acceptable to the francophone countries as they were already operating a structure that could not be implemented in a programme with a shorter duration.

After several consultation among pharmacist educators, the pharmaceutical society of Ghana and regulators (Pharmacy Council, the National Council for Tertiary Education and the National Accreditation Board), the KNUST started a 6-year course to award a Doctor of pharmacy degree in 2012. This was followed by the schools of Pharmacy in the University of Health and Allied Sciences (2017), University of Ghana (2018), University of Development Studies (2018), Central University (2018), and Entrance University College (2018). The minimum entry requirement is a pass in chemistry, biology either physics or elective mathematics from any of these acceptable qualification: West Africa Senior Secondary Certificate Examination (WASSCE), Combined International General Certificate of Secondary Education (IGCSE), Cambridge Advanced Level, International Baccalaureate, General Certificate of Education (GCE), American Grades 12 and 13 examinations and other equivalent external qualifications [26].

PharmD students are trained in basic biomedical, pharmaceutical sciences and pharmacy practice courses and further exposed to longer experiential learning opportunities in both clinical and non-clinical settings. In the first 4 years, students are required to complete 480 h of introductory professional practice in settings such as the community, hospital, industry and regulation [27]. In the final year, students undertake the advance professional practice experiences (APPE) to exposed them to extensive and specialized practical experiences in clinical, hospital, community, regulatory and management as well as industrial pharmacy [27]. A degree in pharmacy is a requirement for licensure examination, therefore both BPharm and PharmD are currently acceptable. The Pharmacy Council is yet to make PharmD the only registrable qualification for licensure [28]. The number of registered pharmacists per year increased over the period as numbers of training institutions increased. Table 3 lists the average number of newly registered pharmacists per decade from 1960 to September 2020.

**Table 3:** Average number of newly registered pharmacists per decade from 1960 to September 9, 2020

Years	Average number (and range) of newly registered pharmacists
1960–1969	7.1 (range 2–17)
1970–1979	23.6 (range 7–44)
1980–1989	30.6 (range 16–38)
1990–1999	62.4 (range 20–121)
2000–2009	103.2 (range 57–137)
2010–2020	222.4 (range 59–411)

Source: Pharmacy Council Ghana, obtained 9th September 2020

**Fig. 1:**
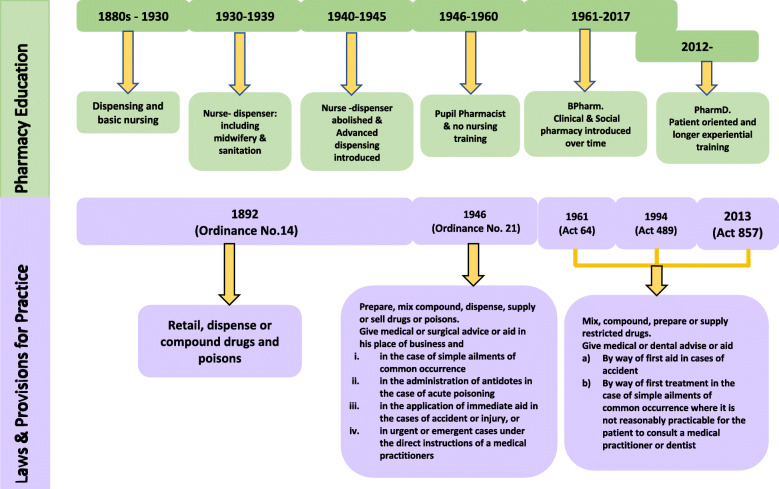
Historical timeline of pharmacy education and legislation for practice

Different policy actors and varied contextual factors had over the years influenced pharmacy education and practice in Ghana from the 1880s to 2017. Table 4 summarizes the policy actors and contextual factors serving as drivers and barriers and the changes made to pharmacy education and practice over time. Pharmacy practice regulation also evolved over the period with commensurate provisions for practice. Figure 1 presents historical timeline of pharmacy education and accompanying legislations for practice.

**Table 4:** Summary of policy actors and contextual factors serving as drivers and barriers and its outcome

Year	Actors, contextual factors serving as drivers	Actors, contextual factors serving as barriers	Outcome: changes in education and practice
1880s - 1930	Actors: Government built hospital (1878) Educators Professional regulators: Board of Examiners Contextual factors: Colonial era. Legislation of the profession (dispensers) through the enactment of Drugs and Poisons Ordinance No.14 of 1892. Government introduced village dispensaries	Contextual factors: Fewer indigenous people with minimum educational background Delayed legislation to reflect training in basic nursing	Education: Training of dispensers introduced Trainee title - Dispenser-in-training Certificate of Dispensing awarded Practice: Dispensing care (compounding and dispensing of prescriptions) and basic nursing in government hospitals and retail shops. Dispensers licensed as Druggists.
1930–1939	Actor: Secretary of State introduced ‘nurse-dispenser’ scheme Educators revised the curriculum Professional regulators: Board of Examiners Contextual factors: High demand for village dispensaries ‘Nurse-dispenser’ scheme already implemented in another British Colony [external context]	Contextual factors: The Drugs and Poisons Ordinance of 1892 not revised to reflect the new role of ‘nurse-dispenser’	Education: ‘Nurse-dispenser’ scheme Curriculum revised Practice: Dispensing care and nursing including midwifery and sanitation in government hospitals, village dispensaries and retail shops. Nurse-dispensers licensed as Druggists
1943–1960	Actors: Medical Department revert to training nurses and dispensers separately Educators revised the curriculum Professional regulators: Pharmacy and Poisons Board Contextual factors: High demand for nurses Legislation of the profession (pharmacists) through the enactment of Pharmacy and Poisons Ordinance No.21 of 1946. Dispensing school relocated to Kumasi College of Science and Technology. Lesser number of pharmacists managing village dispensaries	Under the ‘nurse-dispenser’ scheme most individuals practiced as Druggists Under Pharmacy and Poisons Ordinance No.21 of 1946 *‘No registered pharmacist shall – give medical or surgical advice or aid except in his place of business and –* i. *In the case of simple ailments of common occurrence;* ii. *In the administration of antidotes in the case of acute poisoning;* iii. *In the application of immediate aid in cases of accident or injury, or* iv. *In urgent or emergent cases under the direct instructions of a medical practitioner’*	Education: Curriculum revised Certificate of Competency/ Diploma awarded Trainee title – pupil pharmacist introduced in 1946 Practice: Dispensing care (compounding and dispensing of prescriptions) and reduced role for nursing and medical care in hospitals, retail shop. Licensed as pharmacists.
1961–2017	Actors: Educators revised the curriculum Professional regulators: Pharmacy Board (Act 64) and Pharmacy Council (Act 489) Contextual factors: Kumasi College of Science and Technology gained a university status. Legislation of the profession through the passage of Pharmacy and Drugs Act, 1961 (Act 64) Act 64 repealed and replaced by Pharmacy Act, 1994 (489) National demand for pharmacists with clinical expertise Universities training pharmacists increased		Education: Curriculum revised BPharm degree awarded Department of Clinical and Social Pharmacy established Practice: Experts and advisers on drugs; compounding, preparing and dispensing prescriptions, pharmaceutical care in clinical and non-clinical settings: hospital, community, regulation, academia, industry Licensed as pharmacists
2012 -date	Actors: Educators ECOWAS Ministers of Health Professional regulators: Pharmacy Council Other regulators: National Council for Tertiary Education and National Accreditation Board Pharmaceutical Society of Ghana Contextual factors: Demand for patient oriented pharmacists Pharmacy education evolved globally Global drive to train patient-oriented pharmacists e.g. ECOWAS harmonising pharmacists training Act 489 repealed and replaced by the Health Professions Regulatory Bodies Act, 2013 (Act 857)		Education: Curriculum revised; longer experiential learning in clinical and non-clinical settings introduced PharmD awarded Practice: Experts and advisers on drugs, patient oriented care; compounding, preparing and dispensing prescriptions, pharmaceutical care in clinical and non-clinical settings: hospital, community, regulation, academia, industry Licensed as pharmacists

## Discussion

We have explored the education of pharmacists reforms in Ghana from inception to present. Initially, dispensers were trained in hospitals to assist doctors either in dispensing or directly in diagnosing and treating patients (within limits) in remote facilities. This role has evolved to the present when the pharmacist is a highly trained expert with a strong science base on all aspects of medicines from production and distribution to ensuring responsible use of medicines. Institutional arrangements in terms of training curricula and expected roles in the healthcare system, entry criteria, certificate awarded, and trainee titles have accordingly evolved over time. Contextual factors and policy actors have been important enablers in these reforms.

Contextual factors such as the need for expansion of healthcare service delivery, societal demand for village dispensaries, national laws regulating medical and pharmacy practices, the need to harmonise education of pharmacists across ECOWAS and also internationally served as enablers for government and pharmacy educators to reform pharmacy education. Government representatives such as Secretary of state, governors, medical directors, pharmacy regulators and pharmacist educators have over the years relied on these contextual factors to justify their actions and choices.

Under the four periods studied, different contextual factors presented government and pharmacist educators with evidence and opportunities to push for change in institutional arrangements. For example, with the increased demand for services of dispensers and village dispensaries, the Secretary of State, regulators and educators established a dispensing school and revised the curriculum to train nurse-dispensers to manage village dispensaries. Politically, this was expedient but for a brief period. Less than a decade of implementation, the context of insufficient practicing nurses informed decisions to abolish the nurse-dispenser. Practice and regulatory standards in an interrelating manner informed removal of nursing courses from the pharmacy curriculum. After the nurse-dispenser scheme was abolished and fewer pharmacists operated the village dispensaries, the Medical and Dental Act 1959 [22] reinforced this new reality and legally prevented pharmacists for recovering charges for medical services provided.

A major consideration in these educational reforms has been the international context. Firstly, nurse-dispenser scheme was adopted from Sierra Leone, a former British colony. Also, the term ‘pharmacist’ stated under the Pharmacy and Poisons Ordinance 1946 was adopted from the British Poisons and Pharmacy Act 1908 which restricted the term to persons registered under the Act [29]. Moreover, when the degree programme in pharmacy was introduced at the KNUST, it was aligned with the British curriculum. The trajectory of pharmacy education in Ghana has since been driven by historical experiences, national as well as regional needs and global trends.

Globally, a major determinant of the change in pharmacy education has been the concept of pharmaceutical care. According to Hepler and Strand (1990) ‘*Pharmaceutical care is the responsible provision of drug therapy for the purpose of achieving definite outcomes that improve the patients’ quality of life’* [30]. This has been the major driver of the concept of patient-centered care as opposed to the traditional drug-focused training for pharmacists. In Ghana, this shift has mistakenly been interpreted as pharmacy rediscovering itself to mean a return to the nurse-dispenser who combined rudiments of nursing and pharmacy as a practice. The current practice and roles of pharmacists have expanded to settings including regulation, research, treatment guideline design, academia, pharmaceutical industry, community pharmacy and hospital pharmacy as experts on medicines and providers of pharmaceutical care [8, 31–33].

In other settings, contextual factors have greatly influenced pharmacy educational reforms. According to Supapann et al., 2019, societal needs for specialised skills and expectations served as enablers for educational reforms in Pakistan, South Korea and Thailand. The shift to PharmD training was based on their context and the need to meet demands for pharmacists in clinical settings and in the pharmaceutical industry with enhanced skills [4]. In Saudi Arabia, government actively promoted establishment of training institutions to increase the number of national pharmacists [5]. Changes in pharmacy education in Ghana have similarly been driven by societal and professional introspection.

The training of individuals in dispensing in Ghana initiated from the necessity to support colonial medical officers and this was similar to the case in Nigeria. Pharmacy education in Nigeria is documented to have started in the 1920s and individuals were trained through apprenticeship with a Board of Medical Examiners enacted in 1927 as the regulator [34, 35]. Additionally, the training was rooted in the British educational systems, however, increased societal changes and evolving patients’ needs influenced revisions of curriculum for the pharmacy education in Nigeria [34, 35] and the change in pharmacy education in Ghana have similarly been influenced by societal and patients’ needs.

Globally, there is a wide variation in standards of practice, level of training and capacity of training institutions for pharmacy. The Nanjing conference on pharmacy and pharmaceutical sciences education provided a guide for addressing these disparities. These culminated in the adoption of a global vision for pharmacy where educators are expected to ‘*accept responsibility for the development and sustainability of an adaptable and capable workforce working in partnership for better health care through transformative and continuous education’* [36].

The realities of Coronavirus disease (COVID-19) pandemic confirms the expectations in the FIP statement that *‘Our professional workforce will continuously strive to develop new medicines and to improve the use of existing medicines for better health care. Professional leadership organizations and government agencies can contribute to this vision by supporting progressive policies for professional development and practitioner recognition processes’* [36]. While educators work towards improving the programme to make it truly transformative by reflecting the goals from the Nanjing statements [36], there is the need to build capacities in not only pedagogy but in leadership and advocacy skills to ensure that the impact and expert contributions of pharmacists to health care in Ghana are recognized by policymakers, civil society and patients.

The establishment of the Ghana College of Pharmacists by the Ministry of Health indicates a recognition by government to develop advanced practitioners at specialist and consultant levels capable of demonstrating high level professional skills and expertise. The profession has a responsibility to demonstrate the impact of these changes to health care delivery. To translate these educational transformations to the benefit of society, pharmacists must dedicate themselves to advocate and work with government agencies for support in developing progressive policies for professional development and practitioner recognition process.

The capacity of pharmacists to demonstrate the value of the transformation process is compromised by snail-paced changes in legislation to support newer roles for the profession. There is the need to review the law regulating pharmacy practice to reflect the acquired knowledge and skills as well as allow pharmacists to contribute more to clinical service delivery. Under the Health Professions Regulatroy Bodies Act, 2013, pharmacists can provide ‘*medical and dental advice (a) as first aid where there is an accident or (b) as first aid treatment for simple ailments of common occurrence where it is not reasonably practicable for the patient to consult a medical practitioner or dentist’.* The pharmacist is to refer the patient to a medical practitioner or a dentist after administring the initial dose in the case of an emergency [28]. The law potentially limits the roles and responsibilities of pharmacists in a range of different basic clinical settings. The health system must recognise the current competencies of pharmacists and revise the law accordingly. There is the need to research further and assess the actual roles and responsiblities of new PharmD graduates in the Ghanaian healthcare system.

The number of training institutions and newly registered pharmacists increased over the years in Ghana. However, the pharmacist to population ratio in 2015 was 1:15,000 far below the World Health Organiazation recommendation of 1:2000 [37]. The gap still persist and the June 2020 pharmacist density estimated by the Pharmacy Council is 1.13 per 10,000 population. There is the need to train more pharmacists to bridge this gap.

This study has limitations. The study traced the development and trajectories of education of pharmacists from the 1880s to present. Therefore, data interpretation may be limited by retrospectively mapping and gathering information on important contextual factors influencing the decisions. Colonial reports, correspondents, Ordinances and Acts may mask details of interactions and debates. However, this study relied on multiple data sources and documents based on authencticity [10] to trace historical happenings in a chronological manner to mitigate this limitation.

## Conclusions

Education of pharmacists reforms are context specific and driven by educators, professional bodies and government. As the education of pharmacist journeyed from Dispensing Certificate to Doctor of pharmacy in Ghana, the country’s history, health systems demands, and harmonization with regional and international practice and training were key contextual factors for these reforms. Moreover educators, practice regulators, and professional bodies were able to use these contextual factors as enablers for change. But, these reforms call for direct corresponding change in professional practice laws and regulation to enable pharmacists contribute fully to health care delivery in Ghana. As education of pharmacists and practice constantly reform, we hope this paper contributes to learning beyond Ghana in which the study was conducted to other countries.

## Data Availability

Data sharing is not applicable to this article as no datasets were generated or analysed during the current study.

## Abbreviations

APPE: Advance Professional Practice Experiences
BPharm: Bachelor of pharmacy
ECOWAS: Economic Community of West African States
KNUST: Kwame Nkrumah University of Science and Technology
MPharm: Master of pharmacy
PharmD: Doctor of pharmacy
UG: University of Ghana
WAHO: West Africa Health Organization
WHO: World Health Organization
